# The Mind and the Weather

**Published:** 1894-04

**Authors:** 


### SELECTIONS.

### The Mind and the Weather.

Says Sidney Smith, "very high and very low temperatures establish all human sympathy and relations. It is impossible to feel affection above 78° or below 20° Fahr." Morality is undoubtedly influenced by the state of the thermometer. In India, Hill states 48 per cent. of crimes of violence disappear during cold weather. Lemon, in a recent short article (*Am. Jour. of Psychology*, Jan., 1894), again calls attention to the relation of the weather to psychic states. Among other interesting things he says:

"Certain tendencies are repressed and counter-influences set at work or made dormant. A sudden rise of temperature predisposes those liable to an attack of mania. Some patients are so sensitive to, and dependent on the weather, that they anticipate the thermometer and barometer. One sign of growing neurotic diathesis is inability to keep at the top of one's condition and in good tone in unusual weather. On suicides the effects of the weather are well known. The interesting little book of weather proverbs, published by the weather bureau, is a psychological treasure house upon all this subject.

"Nearly all vocations—some, of course, more than others—are affected by weather. Men of science are often as much subject to weather as seamen. Some writers must have the weather fit the mood, character, or scene, and can do nothing if they are at variance. An adverse temperature brings them to a dead halt. If one will but read poetry attentively, he will be surprised to find how much of it bears weather marks, scattered all through it. A popular writer thinks weather often affects logic, and that many men's most syllogistic conclusions are varied by heat and cold. Diverse weather states may be one cause of so much diversity and even disagreement in thought processes, usually regarded as scientific. I have collected opinions of many experienced teachers, and nearly all think there should be modification of both school work and discipline to correspond with weather. Animals respond to it promptly and with no restraint, and almost constitute a sort of weather signal service if observed. Ancient prognostics were based here. Dunwoody, *e. g.*, gives lists of animals which become restless before rain.

"The fact, that now our property as well as our comfort depend on the weather, has given weather a new power as an excitant which dwellings and clothing had lessened. Man should be joyful or depressed, optimistic or pessimistic over other things than this. The anxiety expressed by the directors of the World's Fair concerning the state of the weather of the opening day was a strain which suggested suffering. Some have said that a certain very famous criminal was acquitted last August solely because the stifling air of the court room made it impossible to follow a severe logical argument. The weather bureau does a great work in economic psychology.

"We have gathered a long list of ejaculatory expressions and unpremeditated remarks concerning weather, which furnish data of interest. Commercial travelers often make much account of it. The head of a factory employing 3,000 workmen said; 'We reckon that a disagreeable day yields about ten per cent. less work than a delightful day, and we thus have to count this as a factor in our profit and loss account.' Accidents are more numerous in factories on bad days. A railroad man never proposes changes to his superior if the weather is not propitious. Fair days make men accessible and generous, and open to consider new problems favorably. Some say that opinions reached in best weather states are safest to invest on.

"Nothing is more curious than the way these ideas have been wrought into description of heaven and hell, whether in heathen, classic, or Christian writings. Hell is very hot or cold, and aggravates pains of all diseases. The *Dies Iræ* was a day of wrath suggested by a day of storm and earthquake.

"Laboratory investigation of the subject meets at the outset the difficulty of distinguishing results of weather changes from similar states otherwise caused. This difficulty is no greater than are many other topics of research, and we believe will not invalidate our methods and results. All our senses put us *en rapport* with the external world. The knee-jerk seems proven to have a weather factor. It is not strange if the eye, *e. g.*, which wants the normal stimulus, in long dark weather causes other changes. Changing moisture in the air changes odors, and many appetites are affected, as touch is still more obviously. Tea-tasters work best on fair days."

Many neurologists have studied with profit the relation of meteorological changes to neuralgia, chorea, and other nervous diseases; and since these morbid conditions of the nervous system seem so easily affected by weather, how much more frequently must the morbid psychic substrata be influenced by barometrical, thermometrical, and electrical states of the atmosphere, and by humidity and the like? But there are many difficulties in the way of investigating the relation of weather to diseased mental conditions. We have noticed among the insane in the asylums exacerbations of mental excitement, the evolution of new delusions, the appearance of novel auditory and visual hallucinations, the development of curious paresthesiæ that alter the kinesthetic perceptions which go to make up personality, under the stimulus of marked meteorological variations. This is a wide field for alienists residing in asylums to explore, and we doubt not that many valuable results would be obtained from a careful, scientific observation of the relations of the weather to psychoses. *American Medico-Surgical Bulletin.*

